# Supplementary material for: Early visual processing and adaptation as markers of disease, not vulnerability: EEG evidence from 22q11.2 deletion syndrome, a population at high risk for schizophrenia
Source: Schizophrenia (Heidelb). 2022 Mar 21;8(1):28. doi: 10.1038/s41537-022-00240-0 (PMC8938446; doi:10.1038/s41537-022-00240-0)
Supplement: Supplementary file 1 — Supplementary material [file 41537_2022_240_MOESM1_ESM.docx]

**Supplementary material**


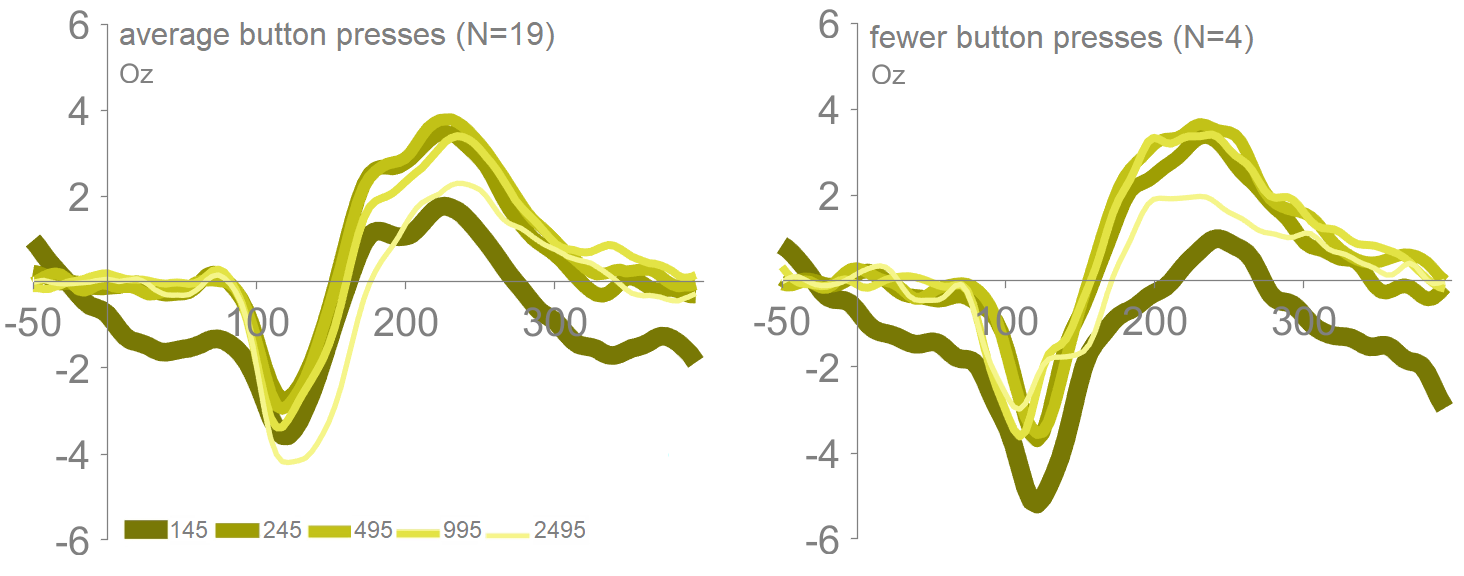


Figure S1. Averaged ERPs per ISI at Oz, showing VEPs for individuals with schizophrenia with average number of button presses versus those with schizophrenia and significantly fewer button presses.


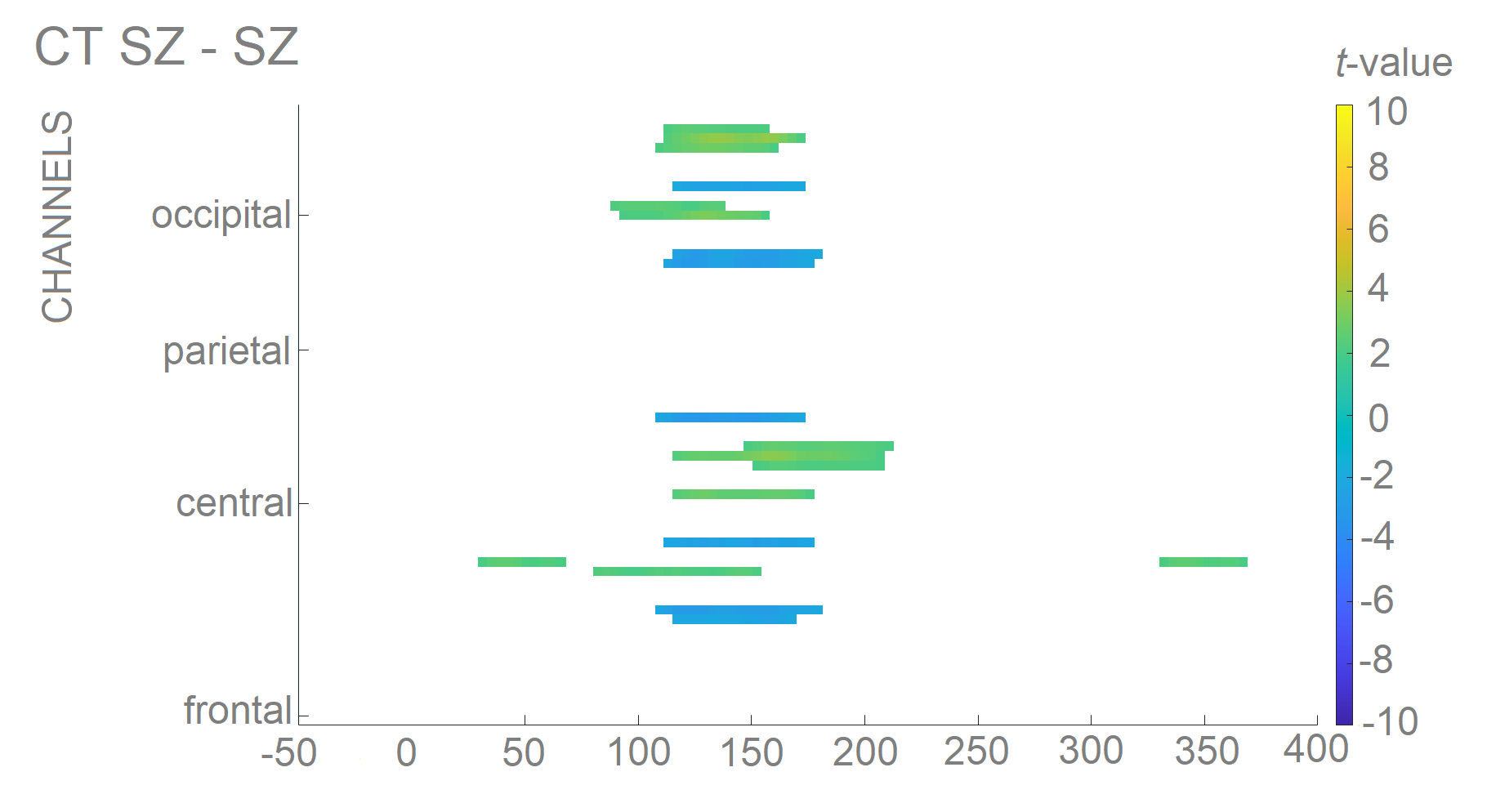


Figure S2. Statistical cluster plot. Color values indicate the p-values that result from point-wise t-tests evaluating the responses across time (x-axis) and electrode positions (y-axis). General electrode positions are arranged from frontal to occipital regions (bottom to top) and the scalp has been divided into four general scalp regions. Within each general region, electrode laterality is arranged from left to right Only p-values <0.05 are color-coded.
